# Developing a team science workshop for early-career investigators

**DOI:** 10.1017/cts.2019.391

**Published:** 2019-07-29

**Authors:** Colleen A. Mayowski, Marie K. Norman, Yael Schenker, Chelsea N. Proulx, Wishwa N. Kapoor

**Affiliations:** Department of Medicine, Institute for Clinical Research Education, University of Pittsburgh School of Medicine, Pittsburgh, PA, USA

**Keywords:** Team science, early-career investigators, clinical and translational science, education, workshop, TL1, KL2, PCOR K12

## Abstract

**Introduction:**

High impact biomedical research is increasingly conducted by large, transdisciplinary, multisite teams in an increasingly collaborative environment. Thriving in this environment requires robust teamwork skills, which are not acquired automatically in the course of traditional scientific education. Team science skills training does exist, but most is directed at clinical care teams, not research teams, and little is focused on the specific training needs of early-career investigators, whose early team leadership experiences may shape their career trajectories positively or negatively. Our research indicated a need for team science training designed specifically for early-career investigators.

**Methods:**

To address this need, we designed and delivered a 2-day workshop focused on teaching team science skills to early-career investigators. We operationalized team science competencies, sought the advice of team science experts, and performed a needs assessment composed of a survey and a qualitative study. Through these multiple approaches, we identified and grouped training priorities into three broad training areas and developed four robust, hands-on workshop sessions.

**Results:**

Attendees comprised 30 pre- and post-doc fellows (TL1) and early-career faculty (KL2 and K12). We assessed impact with a pre- and post-workshop survey adapted from the Team Skills Scale. Results from the pre- and post-test Wilcoxon signed-rank analysis (*n* = 25) showed statistically significant improvement in team science skills and confidence. Open-ended responses indicated that the workshop focus was appropriate and well targeted to the trainees’ needs.

**Conclusions:**

Although team science education is still very much in its infancy, these results suggest that training targeted to early-career investigators improves team skills and may foster improved collaboration.

## Introduction

High impact biomedical research is increasingly conducted by large, transdisciplinary, multisite teams [1,6,7] and funding agencies are prioritizing team science [1–3]. In this increasingly collaborative environment, there is a growing need to ensure that biomedical researchers develop robust teamwork skills. Because these skills are not acquired automatically in the course of traditional scientific education [4], but instead must be fostered deliberately through training and practice [5,6], there have been calls for a concerted focus on team science training [1,7,8]. Team training programs exist; but they tend to focus on teams in clinical settings [9,10] rather than the needs of clinical researchers, whose teams face distinctly different issues [11]. Nor do these existing training programs specifically target the needs of early-career investigators, who face distinctive challenges that are still under explored in the literature [11]. These gaps create an unmet training need for early-career investigators who lead and participate on clinical and translational research teams. To address this need, we designed a 2-day workshop focused on team science skills for early-career investigators. Participants were funded through the University of Pittsburgh’s Clinical and Translational Science Institute (CTSI) and included (a) TL1 pre- and post-doc trainees, who receive rigorous translational research training in the phases of translation (T1-T4); (b) KL2 scholars, who receive individualized, competency-based training in rigorous research methodologies for the design and conduct of high-quality translational research; and (c) PCOR K12 scholars, who receive training in comparative effectiveness research and patient-centered outcomes research (PCOR) methods. This paper describes the design, development, delivery, and assessment of our team science workshop for early-career investigators.

## Background

As the home for premier clinical and translational research training programs (certificate, master’s, and PhD) as well as the Research Education and Career Development Core of the Clinical and Translational Science Institute, the University of Pittsburgh’s Institute for Clinical Research Education (ICRE) is well positioned to offer team science training. Our Team Science Working Group is composed of an experienced clinical researcher and mentor, a science team leader, a teaching and workshop design expert, an adult learning and assessment specialist, and a skilled event coordinator. This personnel combination provided diverse perspectives, content expertise, and skill sets.

## Method

### Literature Review

We began by examining the existing literature on team science. The science of team science (SciTS) integrates research from business and organizational behavior with emerging scholarship on science teams [1,12–16]. SciTS has contributed valuable insights into the issues distinctive to science teams and has offered a range of relevant theoretical models and research-based best practices [1].

Despite its many contributions to our understanding of science teams, SciTS is a young field and still evolving. There remain gaps in the scholarship. During our literature review, we found more focus on large, interdisciplinary, multi-site teams led by established investigators, and less focus on the smaller, less resourced teams of early-career investigators [1]. We also found that the literature emphasized high-level concepts associated with well-functioning teams, such as psychological safety and shared mental models [1,17]. While empirically validated across disciplines and concepts, these concepts were nevertheless theoretical, and, with at least two notable exceptions [7,8], not operationalized for easy application. We also discovered that much of the focus in team science training presumed stable teams, that is, teams with clearly defined membership who could participate in a training together [1], as opposed to investigators in the preliminary stages of team formation.

### Identifying and Operationalizing Competencies

From the literature, we distilled a set of team science competencies. Because “the literature to date has not identified a common set of agreed-on competencies that could serve as targets for design of educational or professional development courses,” [1] we focused on identifying those with specific relevance to our early-career science teams (Appendix 1 in the Supplementary Material). Based on our literature review, we sought to develop training that (a) addressed challenges and circumstances unique to teams led by early-career investigators, (b) was designed to be practical and skills oriented, yet still grounded in theory, and (c) focused on *transportable competencies*: competencies that are applicable to a wide variety of team science contexts, and thus appropriate for investigators whose teams are in flux [18–20].

Two members of our team with backgrounds in educational assessment operationalized these competencies by distilling key themes and metrics in the team science literature, adapted them to early-career investigators, and articulated them at a high level, delineated into specific skills. We arrived at six competency domains subdivided into pragmatic skill areas (Appendix 1 in the Supplementary Material), and used these to inform the development of our training.

### Expert Consultation

We consulted with two internationally known team science experts: Drs. Anita Woolley [21] and Eduardo Salas [22], who helped us identify a set of team science training priorities and models. Woolley and Salas also recommended that we conduct a needs assessment. Using input from our consultants, we developed a needs assessment survey and a qualitative study of teams led by early-career investigators, described below.

### Needs Assessment

#### Survey

We conducted a survey of ICRE KL2, PCOR K12, and TL1 early-career investigators (*n* = 20) to serve as the foundation of our needs assessment (Appendix 2 in the Supplementary Material). In addition to answering several open-ended questions about their experiences on science teams, respondents were asked to rank their level of interest in specific areas of training on a 5-point Likert scale, which we had extracted from the literature review and the operationalized team science competencies (Appendix 1 in the Supplementary Material). The overall level of interest was tabulated by adding the Likert scale responses. For example, if all participants (*n* = 20) rated “Being an effective team leader” a 5, the level of interest would be 100. We used these totals as an indication of our scholars’ overall level of interest in each training topic (Table 1).

**Table 1.** Results of needs assessment survey. Respondents (n = 20) ranked each training need on a 1–5 Likert scale. The overall level of interest was tabulated by adding the Likert scale responses

Training need	Level of interest
Being an effective team leader	86
Holding team members accountable	86
Clarifying roles and expectations	79
Developing shared goals	77
Providing feedback to team members	76
Maintaining team morale	76
Managing people	76
Managing conflict	75
Assessing team performance	74
Dealing with setbacks	72
Maintaining mutual respect	72
Delegating responsibilities	71
Recruiting, interviewing, and hiring staff	70
Managing change	67
Recruiting the right collaborators	66

*Note*. Survey participants identified other issues, including negotiating authorship, finding funding, and communicating across disciplines, but the total scores did not merit inclusion in this table.

#### Qualitative study

Now that we had operationalized competencies, consulted with team science experts, and conducted a needs assessment, we next conducted a qualitative study of teams led by early-career investigators in our KL2 and PCOR K12 programs to better understand the challenges identified by our trainees. Our qualitative study is thoroughly described elsewhere [11], but briefly, we found that while the teams of early-career investigators reported high levels of trust and strong communication, there were also underlying tensions on teams that, if left unaddressed, could adversely affect effective team functioning. This study suggested a number of desired skill sets and potentially productive training areas (particularly pertaining to goal-setting, management of scientific disagreements, establishment of team boundaries, and the transition to leadership); it also reinforced the advice of our consultants and provided us with richly contextualized scenarios to inform our training. Taken together, our qualitative study [11] and our needs assessment (Appendix 2 in the Supplementary Material) reinforced the need for highly practical skills training in areas such as finding collaborators, hiring staff, running team meetings, and managing conflict.

## Workshop Development

Using expert consultations, the needs assessment survey, the qualitative study, and the team science literature, we developed “Building Successful Research Teams,” a 2-day workshop for early-career investigators focused on practical skill acquisition and immediate application.

### Organization

We grouped the training priorities indicated by survey respondents into three broad training areas (knowledge, skills, and attitudes) and then developed four 3-hour sessions delivered over 2 days. The sessions were Characteristics of Effective Teams; Recruiting and Evaluating a Team; Leading Meetings Effectively; and Managing Difficult Conversations on Teams. These sessions were facilitated by members of the Team Science Working Group as well as expert educators who were themselves experienced collaborators and researchers. Attendance at the workshop was a required component of the ICRE’s team science training for TL1, KL2, and PCOR K12 trainees. Other CTSA T-and-K training programs (for example, T32s) were intentionally not included because of space limitations and the facilitators’ preference for limiting size to more effectively implement active discussions and small group exercises.

We categorized the knowledge, skills, and attitudes we wanted trainees to gain in each of the four sessions (Table 2), drawing on the high-level competencies we had identified (Appendix 1 in the Supplementary Material).

**Table 2.** Knowledge, skills, and attitudes vital to building successful research teams

	Characteristics of effective teams	Forming teams	Running productive meetings	Giving effective feedback and managing conflict
Knowledge	Discuss the importance of teams and teamwork Identify the unique challenges of science teams	Discuss issues, priorities, and challenge in team formation	Identify best practices and common pitfalls in running meetings	Identify the characteristics of effective feedback Identify best practices in giving (and receiving) feedback
Skills		Approach collaborators Evaluate applicants Conduct interviews	Plan a meeting and build an agenda Analyze well and poorly run meetings Begin a meeting effectively End a meeting effectively	Respond appropriately to problematic behaviors Give constructive feedback in the moment Give constructive feedback 1-on-1
Attitudes	Appreciate the benefits of collaboration and cognitive diversity			

Each session combined research-based content with opportunities for discussion and hands-on practice through group exercises, case analyses, and role-playing. We integrated adult learning principles by drawing on trainees’ prior experiences and by keeping the material concrete, problem-based, and solution-focused. (See Appendix 3 in the Supplementary Material for a detailed description of each session.) Throughout all the sessions, we incorporated opportunities for reflection and planning, thus encouraging transfer of learning to trainees’ work situations.

## Assessment

We assessed the impact of “Building Successful Research Teams” with a pre- and post-workshop survey (Appendix 4 in the Supplementary Material). We adapted the 17-item Team Skills Scale [25], adapting the language slightly to shift the emphasis from clinical to research scenarios. Our adapted Team Skills Scale asked participants to rate their ability to carry out a series of team-skills-related tasks. To this, we added questions 18–26 (Appendix 4 in the Supplementary Material), generated by workshop session leaders, which asked participants to rate their confidence in handling topics covered during the workshop. The pre-test was administered online at the time of registration; the post-test was made available to attendees online at the end of the 2-day workshop. The post-test differed slightly from the pre-test in that it included questions about satisfaction unrelated to content, such as the workshop space. The post-test also included questions about how participants planned to apply what they learned to their research practice. We checked for significance between pre- and post-test responses to Items 1-26 using the Wilcoxon signed-rank test.

## Results

We conducted “Building Successful Research Teams” on April 5 and 6, 2018. It was held off-site, to encourage trainees’ full immersion, and was facilitated by members of the Team Science Working Group as well as expert educators who were themselves experienced collaborators and researchers. The attendees (*n* = 30) were composed of 18 pre- and post-doc TL1, 8 KL2, and 4 PCOR K12 trainees. Their clinical and translational research interests spanned disciplines of bioengineering, medicine, nursing, and public health.

Table 3 presents analysis of pre- and post-workshop responses to the adapted Team Skills Scale [25] (questions 1–17, Appendix 4 in the Supplementary Material). Table 4 presents analysis of pre- and post-workshop responses to questions focused on self-reported confidence in their ability to apply team science skills (questions 18–26, Appendix 4 in the Supplementary Material). Results from the pre- and post-test analysis (*n* = 25; 83%) showed statistically significant improvement for every question except the two highest rated items on the pre-test: Item 2 (the ability to treat team members as colleagues) and Item 8 (the ability to carry out responsibilities specific to their role on the team).

**Table 3.** Adapted team skills scale: differences between pre- and post-workshop test scores (n = 25 attendees)

Items	Pre-test			Post-test			Wilcoxon Z
	Median	Mean	St. Dev.	Median	Mean	St. Dev.	
Team skills scale composite score	58	57.36	8.67	70	68.80	10.24	−**4.04** ^***^
Function effectively in an interdisciplinary team	4	3.88	0.67	4	4.16	0.55	−**2.33** ^*^
Treat team members as colleagues	4	4.28	0.61	5	4.40	0.76	−0.83
Identify contributions to research that different disciplines can offer	4	3.56	0.92	4	4.20	0.76	−**3.21** ^**^
Apply your knowledge of research methods in a team setting	4	3.64	0.91	4	4.24	0.72	−**3.35** ^***^
Ensure that team members’ preferences/goals are considered when working together	4	3.40	0.71	4	4.16	0.85	−**3.09** ^**^
Handle disagreements effectively	3	2.88	0.93	4	3.68	0.90	−**3.30** ^**^
Strengthen cooperation among disciplines	3	3.28	0.79	4	4.00	0.76	−**3.49** ^***^
Carry out responsibilities specific to your role on a team	4	4.16	0.55	4	4.36	0.64	−1.51
Address issues succinctly in team meetings	3	3.32	0.80	4	3.96	0.73	−**2.81** ^**^
Participate actively at team meetings	4	3.68	1.11	4	4.24	0.93	−**2.07** ^*^
Develop a collaboration agreement	2	2.28	1.02	4	3.32	1.03	−**3.57** ^***^
Adjust your approach to support the team goals	4	3.44	0.82	4	4.04	0.89	−**2.44** ^*^
Develop strategies that help your team attain research goals	3	3.40	0.76	4	4.20	0.76	−**3.68** ^***^
Raise appropriate issues at team meetings	3	3.36	0.76	4	4.08	0.86	−**3.36** ^***^
Recognize when the team is not functioning well	4	3.40	0.96	4	4.24	0.66	−**3.55** ^***^
Intervene effectively to improve team functioning	3	2.76	0.83	4	3.76	0.88	−**3.84** ^***^
Help draw out team members who are not participating actively in meetings	3	2.64	0.86	4	3.76	0.88	−**3.99** ^***^

*Note*. Item scales range from 1 (poor) to 5 (excellent). Range of possible values for composite scale score is 17–85. Bolded values indicate significance.

*P < 0.05, ^**^P < 0.01, ^***^P < 0.001.

**Table 4.** Self-reported confidence in team skills, pre- and post-workshop (n = 25 attendees)

Items	Pre-test			Post-test			Wilcoxon Z
	Median	Mean	St. Dev.	Median	Mean	St. Dev.	
Explain why science is increasingly conducted in teams	4	3.68	1.03	5	4.68	0.48	−**3.67** ^***^
Identify the characteristics of effective science teams	3	3.32	1.07	4	4.32	0.63	−**3.20** ^**^
Spot a “red flag” in an applicant’s CV	2	2.32	0.90	4	3.96	0.84	−**4.15** ^***^
Get the information I need when checking an applicant’s references	2	2.40	0.96	4	3.84	0.69	−**4.08** ^***^
Assemble a team composed of the right people	3	2.64	0.91	4	3.80	0.96	−**3.78** ^***^
Run an effective team meeting	3	3.04	0.84	4	4.04	0.68	−**3.62** ^***^
Avoid common team meeting pitfalls	3	2.68	0.95	4	4.08	0.64	−**4.23** ^***^
Give effective feedback “in the moment”	3	2.64	0.95	4	3.80	0.76	−**3.94** ^***^
Constructively address problematic team behavior	2	2.40	1.04	4	3.76	0.72	−**3.94** ^***^

*Note*. Item scales range from 1 (not at all confident) to 5 (very confident). Bolded values indicate significance.

^**^P < 0.01, ^***^P < 0.001.

We also asked several open-ended questions (numbers 27 through 36 in Appendix 4 in the Supplementary Material) including, “Which part of the workshop did you find most useful, and why?” Forty-four percent of the participants responded that the moderated panel discussion was most useful. Participants also highlighted the sessions on running effective team meetings and having difficult conversations as common concerns that were well addressed during the workshop. When responding to why the workshop was helpful, 60% mentioned its practical focus, while the other 40% mentioned the experienced and diverse advice presented.

We requested and received suggestions for improvement. Twenty-four percent of participants felt that 2 days was too long; they suggested the content could have been condensed into 1 day. A few of participants (those in the earliest stages of their careers) felt that the information presented about hiring effective team members and evaluating CVs were not yet applicable to their career stage.

Participants were also asked to list two things that they would do differently as a result of the workshop. The comments focused on practical takeaways including “Craft better meeting agendas and conduct better reference checks for new hires,” “Approach difficult conversations from a helping/understanding perspective,” and “Try to focus on creating shared meaning when giving feedback.”

## Discussion/Conclusion

We developed and ran a 2-day team science workshop for early-career investigators focused on building knowledge, skills, attitudes, and confidence in the topic areas shown in Table 2 and Appendix 3 in the Supplementary Material.

The post-workshop assessment indicated that attendees felt better prepared to engage in key team science activities after participating. Only two items did not reach significance, and given that these two items were the most highly rated at baseline, this is not surprising.

While we believe the workshop met its goals, we also believe that future iterations could be improved. Developing, delivering, and assessing the workshop left us with the lessons and takeaways discussed below.

First, training should be customized for scholars at different career stages. Although our concrete, practical focus was appropriate and the content was well targeted to our trainees’ needs, its applicability sometimes depended on the trainee’s career stage. TL1 trainees, for example, were for the most part not yet leading research teams and thus had more difficulty than KL2 scholars when connecting their experiences to that workshop content. In the future, we may consider offering customized workshops for scholars at different career stages.

Second, training should focus more on hands-on activities and opportunities to engage in dialog with experienced team leaders as opposed to didactic content. Our evaluation reinforced our research that participants preferred practical, skills-based content to didactic content. In particular, attendees appreciated hearing from and asking questions of science team leaders as they described the pitfalls and rewards of leading teams during the panel discussion. Guided practice in designing agendas and evaluating CVs, and other activities, like role-playing difficult conversations, were highly rated by participants. Based upon this feedback, we will look for ways to shorten the didactic components of the workshop and include more hands-on activities and opportunities to learn from experienced team leaders.

Third, the workshop should be shortened. A 2-day workshop may be too long for trainees who are balancing research training with clinical responsibilities. In response, we plan to shift the format from a 2-day intensive workshop to a series of shorter, targeted workshops spread out over the year, each focused on a practical skill set. For example, throughout 2018–2019, we are offering 2-hour workshops on Budgets and Project Management, Managing Up, and Use of Technology in Teams and Research. We are also re-imagining our content and recreating activities that will translate well to a series of online modules for greater scalability and wider dissemination. These modules will have a flexible design to allow for fully online or hybrid implementation, with self-paced activities, reflective exercises, discussion questions, and optional assignments to promote skill building and knowledge transfer.

We hope our experience informs others as they craft team science training for early-career investigators. However, team science education is still very much in its infancy. Thus, it is particularly important that institutions embarking on training initiatives share their approaches, results, and lessons learned, evaluating them carefully, and assessing their applicability for different trainee populations and institutional needs. While the process described in this paper was used successfully at a single institution and the outcomes may not be fully generalizable, the consistency of our approach and results with the broader research on team science training suggests that it might be an approach worth replicating.

## Supplementary material

For supplementary material accompanying this paper visit https://doi.org/10.1017/cts.2019.391. click here to view supplementary material
