# Supplementary material for: Developing a team science workshop for early-career investigators
Source: J Clin Transl Sci. 2019 Jul 29;3(4):184–9. doi: 10.1017/cts.2019.391 (PMC6799325; doi:10.1017/cts.2019.391)
Supplement: Supplementary file 1 [file S2059866119003911sup001.pdf]

Appendix 1: Operationalized Team Science Competencies

1. Team Formation: Use recommended recruitment and hiring methods to assemble a team with appropriate skills and knowledge, compatible working styles, and sufficient availability.

2. Team Communication: Define a vision, align goals, clarify roles and responsibilities, and set clear expectations. Encourage a range of perspectives and address conflict proactively.

3. Team Orientation: Prioritize team meetings, meet deadlines, contribute regularly, take responsibility for moving the team forward, and demonstrate a positive, collaborative outlook.

4. Team Management: Coordinate team members, distribute work appropriately, hold regular meetings with clear goals and agendas, plan thoughtfully, and set realistic time tables and deadlines.

5. Team Assessment: Establish metrics to gauge success, monitor team and individual progress, provide regular feedback to team members, and solicit feedback on your own performance.

6. Team Morale: Build relationships and establish trust, provide motivation for sustained engagement, reward contributions, and give recognition fairly.

Appendix 2: Needs Assessment Survey

We'd be very grateful if you would take 5 minutes to complete this short survey. Your responses will be strictly confidential, so please answer honestly. Your participation is voluntary.

Are you: TL1 ☐ KL2 ☐ K12 ☐ Other ☐

Question 1: Think about a particularly effective team you've been part of. What are the characteristics of the team that made it effective?

Question 2: Think about a particularly ineffective team you've been part of. What are the characteristics of the team that made it ineffective?

Question 3: Please indicate your level of interest in each of the following team training areas from <u>not very interested</u> (1) to <u>very interested</u> (5)

	1	2	3	4	5
Recruiting the right collaborators					
Recruiting, interviewing, and hiring staff					
Delegating responsibilities					
Managing people					
Holding team members accountable					
Being an effective team leader					
Developing shared goals					
Clarifying roles and expectations					
Providing feedback to team members					
Assessing team performance					
Maintaining mutual respect					
Managing conflict					
Dealing with setbacks					
Maintaining team morale					
Managing change					
Other areas of team training:					

Thank you for taking the time to complete this survey.

Appendix 3: Session Descriptions

Session 1: We began the first session with TEDTalk speaker Tom Wujec's marshmallow challenge exercise, which asks participants to work in teams to accomplish a time and resource-constrained (though fun) task. We debriefed the exercise to highlight features of effective and ineffective teams, and showed Wujec's "Build a Tower, Build a Team" TED Talk[24] to introduce additional facets to the conversation. This was followed by an interactive didactic session on the growth of team science, the reasons for this growth, and what the research says about the characteristics of effective teams. Along the way, participants were asked to reflect on their own experiences working in teams.

Session 2: In this session, we focused on how to assemble a high-functioning team. The presenter discussed several relevant models; then, we asked a panel of investigators at varying career stages to engage in a moderated discussion around hiring and other HR issues. Participants were given a set of resumes that exemplified common issues (e.g., gaps in employment, sloppy mechanics, over-qualified job candidates) as well as distinctions (e.g., evidence of initiative, awards) and discussed their relative merits, red flags, etc. in small groups.

Session 3: This session dealt with one of the issues that emerged most frequently in our needs assessment: team meetings. For this session, we created a set of video cases (starring our own faculty) which depicted team meetings done well and poorly. After watching each video case, participants analyzed what they observed and discussed a range of issues including the importance of creating and distributing agendas, time management, celebrating successes, managing difficult personalities, and managing up. These videos borrowed directly from issues

raised in our needs assessment and qualitative research, which made the content relevant and engaging.

Session 4: In this session, we discussed techniques and strategies for engaging in difficult conversations and giving feedback using the Crucial Conversations[25] framework. Participants were given a set of short cases describing problematic situations and were asked to role-play conversations addressing those situations. Again, the scenarios were drawn from issues identified in the survey and qualitative research.

Appendix 4: Building Successful Research Teams Post-Test. The post-test differed only slightly from the pre-test in that it included satisfaction questions and questions regarding how the participants might change their practice.

Team Science 2-Day Workshop
April 5 & 6, 2018
Post-Workshop Survey

Please complete the following survey before you leave today. Your responses are anonymous.

Please rate your ability to carry out each of the following tasks:

	Poor	*Fair*	*Good*	*Very Good*	*Excellent*
1. Function effectively in an interdisciplinary team	o	o	o	o	o
2. Treat team members as colleagues	o	o	o	o	o
3. Identify contributions to research that different disciplines can offer	o	o	o	o	o
4. Apply your knowledge of research methods in a team setting	o	o	o	o	o
5. Ensure that team members' preferences/goals are considered when working together	o	o	o	o	o
6. Handle disagreements effectively	o	o	o	o	o
7. Strengthen cooperation among disciplines	o	o	o	o	o
8. Carry out responsibilities specific to your role on a team	o	o	o	o	o
9. Address issues succinctly in team meetings	o	o	o	o	o
10. Participate actively at team meetings	o	o	o	o	o
11. Develop a collaboration agreement	o	o	o	o	o
12. Adjust your approach to support the team goals	o	o	o	o	o
13. Develop strategies that help your team attain research goals	o	o	o	o	o
14. Raise appropriate issues at team meetings	o	o	o	o	o
15. Recognize when the team is not functioning well	o	o	o	o	o
16. Intervene effectively to improve team functioning	o	o	o	o	o

17. Help draw out team members who are not participating actively in meetings	o	o	o	o	o

Hepburn, Tsukuda, and Fasser (1996), Team Skills Scale, all rights reserved

Likert Scale, 1-5, Not at all confident to Very Confident
1=Not at all confident
3=Somewhat confident
5=Very confident

Please rate your confidence in your ability to:

18. Explain why science is increasingly conducted in teams.
19. Identify the characteristics of effective science teams.
20. Spot a "red flag" in an applicant's CV.
21. Get the information I need when checking an applicant's references.
22. Assemble a team composed of the right people.
23. Run an effective team meeting.
24. Avoid common team meeting pitfalls.
25. Give effective feedback "in the moment."
26. Constructively address problematic team behavior.

Team Science 2-Day Workshop
April 5 & 6, 2018
Workshop Evaluation Form

Your feedback will help us improve this workshop. Please take a few minutes to share your opinions.
Please turn in your evaluation today. Thank you!

	Strongly disagree				Strongly agree
27. The content was applicable to my role	1	2	3	4	5
28. I would recommend this workshop to others	1	2	3	4	5
29. The material was presented in an organized manner	1	2	3	4	5
30. I would be interested in attending a follow-up, more advanced workshop on team science	1	2	3	4	5

31. Given the topic, was this workshop: ❑ Too short ❑ Right length ❑ Too long

32. Please rate the following:

	Excellent	Very Good	Good	Fair	Poor
a. Group exercises	❑	❑	❑	❑	❑
b. The program overall	❑	❑	❑	❑	❑
c. Didactic content	❑	❑	❑	❑	❑
d. Handouts/Take home materials	❑	❑	❑	❑	❑

33. Which part of the workshop did you find most useful and why?

34. Which part of the workshop was the least helpful and why?

35. List two things you will do differently as a result of this workshop.

36. Please provide any additional comments.

Thank you!
